# Syphilis notifications among pregnant women in Campo Grande, state of Mato Grosso do Sul, Brazil, 2011 to 2017

**DOI:** 10.1590/0037-8682-0024-2020

**Published:** 2020-09-11

**Authors:** Cássia de Paula Pires, Caroliny Oviedo Fernandes, Everton Falcão de Oliveira, Sandra Luzinete Felix de Freitas, Rodrigo Guimarães dos Santos Almeida

**Affiliations:** 1 Universidade Federal de Mato Grosso do Sul, Programa de Pós-Graduação em Doenças Infecciosas e Parasitárias, Campo Grande, MS, Brasil.; 2 Universidade Federal de Mato Grosso do Sul, Programa de Residência em Enfermagem Obstétrica, Campo Grande, MS, Brasil.; 3 Universidade Federal de Mato Grosso do Sul, Instituto Integrado de Saúde, Campo Grande, MS, Brasil.

**Keywords:** Treponemal infections, Antenatal care, Treponema pallidum, Syphilis, Congenital syphilis

## Abstract

**INTRODUCTION:**

Syphilis is a sexually transmitted infection caused by *Treponema pallidum*. Considering the high rates of syphilis in pregnancy and congenital syphilis reported in Brazil in the past, and their serious consequences, this study described the epidemiological and clinical profile of pregnant women with a confirmed diagnosis of syphilis in Campo Grande, in the state of Mato Grosso do Sul, Brazil, from 2011 to 2017.

**METHODS:**

This is a descriptive study, based on syphilis notifications among pregnant women reported to the *Sistema de Informação de Agravos de Notificação* (National System of Disease Notification of Brazil).

**RESULTS:**

Over the study period, 2,056 confirmed cases of syphilis in pregnancy were reported, resulting in a crude cumulative incidence of 144.76 cases per 1,000 live-born babies. The incidence increased from 9.97 cases per 1,000 live-born babies in 2011 to 36.10 cases per 1,000 live-born babies in 2017. It was more prevalent in women who were young, of mixed race, with low educational attainment. Over one third of women were diagnosed in the first trimester of pregnancy; therefore, they were at risk of reinfection if they or their sexual partners were inadequately treated. Furthermore, syphilis was not well classified according to its clinical stage, which led to inappropriate treatments.

**CONCLUSIONS:**

Despite efforts to reduce the incidence of syphilis, syphilis during pregnancy remains a public health problem, reflecting possible inadequacies in antenatal care, especially in vulnerable populations. It is important to include sexual partners in syphilis treatment during pregnancy to prevent reinfection.

## INTRODUCTION

Syphilis is an infectious disease caused by *Treponema pallidum*, which has cutaneous and systemic manifestations. Treponemal and non-treponemal tests are used in diagnosis, and benzathine penicillin is the standard treatment. There are four stages of infection: primary, secondary, tertiary, and latent. The required dose and duration of antibiotic treatment depends on the stage: the more advanced the stage, the greater the required dose^1^ ^,^ ^2^.

According to the World Health Organization, syphilis affects more than 12 million people worldwide, with 2 million pregnant women infected annually^3^. Syphilis in pregnancy can cause congenital syphilis, which may have serious health consequences.

Congenital syphilis is a disease of public health importance because it can cause early and late complications in infants, including premature birth, low birth weights, skin lesions, periostitis or osteitis, anemia, and seizures^4^. In 2013, congenital syphilis caused more than 300,000 fetal and neonatal deaths, and more than 215,000 childhood deaths worldwide^2^. The incidence rate of congenital syphilis in Brazil was 9.0 cases per 1,000 live births in 2018^3^. In 2016, the *Plan of Action for the Prevention and Control of HIV and Sexually Transmitted Infections (2016-2021)* was released, and prevention measures, including increased access to diagnosis and early treatment of HIV infection and other sexually transmitted infections (STIs), were expanded to reduce their incidence by 2021^5^.

The persistently high incidence of syphilis is a major public health problem in Brazil, with serious consequences^2^. Management of the consequences of this disease, especially the tertiary form in adults and congenital syphilis, is costly in terms of healthcare resources.

This study aimed to estimate the incidence of syphilis in pregnancy as well as congenital syphilis, and to describe the epidemiological and clinical-obstetric features of the reported and confirmed cases of syphilis in pregnant women in Campo Grande, Mato Grosso do Sul State, Brazil.

## METHODS

We conducted a descriptive and retrospective study based on syphilis cases in pregnant women and congenital syphilis reports from the *Sistema de Informação de Agravos de Notificação* (National System of Disease Notification of Brazil - Brazilian abbreviation: SINAN) in Campo Grande, from 2011 to 2017. All data were provided by the Municipal Secretary of Public Health of Campo Grande.

All cases of syphilis in pregnant women in Campo Grande that were confirmed using treponemal and/or non-treponemal tests, who also did not have a previous treatment record, were included. Cases reported outside the study period, cases of syphilis in pregnant women living in other municipalities or in rural and peri-urban areas, and women who were seropositive from a previous infection were excluded.

The data included age, racial origin, marital status, level of education, the trimester of diagnosis, clinical data on diagnosis and treatment of the pregnant women (including clinical classification, treponemal and non-treponemal test results, and the treatment provided); it also included data on treatments received by sexual partners (treatment completion, therapy used, and reason for non-treatment), and infants born to women with syphilis during pregnancy.

Analyses were performed using Statistical Package for Social Sciences version 22 (IBM Corp., Armonk, NY, USA).

The study protocol was approved by the Universidade Federal de Mato Grosso do Sul Research Ethics Committee (CAAE: 69740517.8.0000.0021; protocol: 2.166.457), which granted a waiver of the requirement for consent, and the study was conducted in accordance with the principals of the Declaration of Helsinki.

## RESULTS

During the study period, 2,255 cases of syphilis in pregnant women were notified and recorded in SINAN. After applying the exclusion criteria, 2,056 cases were included in the analysis. The highest syphilis frequency in 2017 was almost 4 times higher than that in 2011, and the crude incidence cumulative per year continuously increased (Table 1).

**TABLE 1:** Frequencies of live-born, reported cases of syphilis diagnosed in pregnant women, and the crude incidence per 1,000 live-born babies, according to the year (N = 2,056).

Year	Population (live births)	Reported cases	Incidence (1000 inhabitants)
2011	13,045	130	9.97
2012	13,539	193	14.26
2013	13,693	249	18.18
2014	14,203	243	17.11
2015	14,470	328	22.67
2016	13,728	398	28.99
2017	14,264	515	36.10

The incidence was highest in women in the 20-29 years age group, followed by those in the 10-19 years age group (Table 2). More than half of the cases occurred among women of mixed race, and the incidence was higher among women with less than 10 years of schooling than among women with a higher level of education. Over one third of the women were in the first trimester of pregnancy at the time of diagnosis (Table 2).

**TABLE 2:** Sociodemographic characteristics of women reported with syphilis during pregnancy (N = 2,056).

Variables	N	%
**Age (years)**		
10 to 19	533	25.9
20 to 29	1034	50.3
30 to 39	435	21.2
≥40 years	54	2.6
**Race**		
Asian	16	0.8
Indigenous	23	1.1
Black	129	6.3
White	605	29.4
Mixed	1160	56.4
Missing/unreported	123	6.0
**Age at completing schooling**		
<10 years old	843	41.0
From 10 to 12 years old	700	34.0
>12 years old	57	2.8
Missing/unreported	456	22.2
**Trimester of infection**		
First	714	34.7
Second	617	30.0
Third	556	27.0
Missing/unreported	169	8.3
**Region of residence**		
Anhanduizinho	699	34.0
Bandeira	286	13.9
Centro	49	2.4
Imbirussu	218	10.6
Lagoa	344	16.7
Prosa	167	8.1
Segredo	282	13.7
Missing/unreported	11	0.6

Of the women with information on the clinical stage at the time of diagnosis, the majority were in the tertiary or latent phase, and the majority of infections were confirmed by treponemal tests (Table 3).

In order to consider a pregnant woman as having been adequately treated in this study, treatment should be terminated within 30 days before delivery, should occur concurrently with the sexual partner's treatment, and the dose of benzathine penicillin should be appropriate for the clinical phase^2^. Based on these criteria, treatment was considered adequate in less than half of pregnant women (Table 3).

**TABLE 3:** Clinical characteristics and treatment of women reported with syphilis during pregnancy (N = 2,056).

Variants	N	%
**Clinical stage**		
Primary	354	17.2
Secondary	40	1.9
Tertiary	590	28.7
Latent	510	24.8
Missing/unreported	562	27.3
**Treponemal test**		
Reactive	1872	91.1
Non-reactive	29	1.4
Missing/invalid	155	7.5
**Non-treponemal test**		
Reactive	1254	61.0
Non-reactive	260	12.6
Missing/invalid	542	26.4
**Treatment of pregnant woman**		
Appropriate	1006	48.9
Inappropriate	1050	51.1

The largest shortcoming in the management of syphilis among women in the study was the diagnosis and treatment of the sexual partners of women reported with syphilis during pregnancy. Diagnostic tests and treatment were not performed in almost half of the partners. Therefore, their treatment could be considered inadequate as well as the lack of contact with the pregnant woman and partners who did not attend the health unit when requested (Table 4).

**TABLE 4:** Clinical characteristics and treatment of sexual partners of women reported with syphilis during pregnancy (N = 2,056).

Variants	N	%
**Test performed**		
Yes	1056	51.4
No	873	42.5
Missing/ unreported	127	6.2
**Therapeutic scheme**		
Appropriate	569	27.5
Inappropriate	803	38.9
Unperformed	684	33.1
**Reasons for non-treatment of the partner**		
Lack of contact with the pregnant woman	365	17.8
Partner did not attend	135	6.6
Partner’s identity was not reported	16	0.8
Partner had a non-reactive test result	66	3.2
Partner refused	50	2.4
Other	264	12.8
Missing	1160	56.4

A total of 907 cases of congenital syphilis were reported among the 2,056 women reported with syphilis during pregnancy in Campo Grande from 2011 to 2017. Thus, 44.1% of women with syphilis diagnosed during pregnancy had an unfavorable outcome with serious consequences for their babies, such as miscarriages, stillbirths, prematurity, and other clinical manifestations^2^.

## DISCUSSION

During the study period, there was a continuous and marked increase in the incidence of syphilis diagnosed during pregnancy. Considering the introduction of mandatory notifications of gestational syphilis in Brazil in 2005, this continuous increase may result in improvements in the reporting system due to better human resource capacities, a pregnant women’s increased access to prenatal consultations as a result of the implementation of Family Health Strategy teams, and increased epidemiological surveillance. Such actions are aimed at improving women’s health during antenatal care in order to achieve a reduction in gestational and congenital syphilis^6^ ^,^ ^7^. However, other possible causes must be considered, such as those related to behavioral issues^8^.

Furthermore, there was a relatively high number of cases of syphilis diagnosed in pregnant adolescents. This is a cause of concern for health services because women reach reproductive age during adolescence. In addition, the number of cases of syphilis diagnosed in pregnant adolescents increased over the study period. In this study, adolescents accounted for 25.9% of the total number of cases of syphilis diagnosed during pregnancy. In other studies of syphilis among pregnant women conducted in Brazil between 2010 and 2014, adolescents accounted for less than 20% of cases of syphilis diagnosed during pregnancy^9^ ^,^ ^10^ ^,^ ^11^.

Studies have shown that people of mixed race and a lower level of education have a higher incidence of STIs. This has been attributed to a lack of information about prevention and treatment, and a high incidence of unprotected sex among women in these demographic groups^12^ ^,^ ^13^. Macêdo et al. showed that the incidence of syphilis during pregnancy was higher among women of low socioeconomic status and among those who received inadequate antenatal care^14^. In addition, unsafe sexual practices and lack of social support have been shown to increase the risk of recurrent infections and reinfection^14^.

The determinants of syphilis and congenital syphilis are not only related to the quality of antenatal care offered to women, but are rooted in the social, economic, cultural, and behavioral factors that women experience. During pregnancy, early diagnosis is critical in order to reduce the risk of vertical transmission. This is because in the early stages of infection there is a higher number of circulating spirochetes and a higher risk of transmission to the fetus¹.

In terms of the clinical stages seen in this study, the majority of the women were in the tertiary or latent phase. This differs from the frequencies reported in other studies, where most cases were in the primary phase^15^ ^,^ ^16^. In these other studies, the pregnant women might have been classified incorrectly, because if screening were conducted, a higher proportion of women would be expected to be in the latent phase^17^. In addition, the more advanced the clinical stage, the greater the dose and duration of treatment that is required. Therefore, it is important that healthcare providers establish a good relationship with primary care users in order to minimize loss to follow-up,^18^ and to ensure that those who require prolonged treatment, particularly those with tertiary or latent syphilis, complete their full course of treatment. However, there were many women with missing data due to a failure on the part of the healthcare providers to complete all items on the notification form. This may have compromised the quality of the data.

Treponemal tests were used increasingly. Although confirmation of the diagnosis using treponemal tests is important, ideally treponemal tests should be performed together with a non-treponemal test. This makes it possible to correctly classify the clinical phase of the disease so that the appropriate dose and duration of treatment can be administered^1^.

Most of the women in this study received inadequate treatment. This is a concern because a lack of adequate treatment increases the risk of vertical transmission. Although the proportion of women who received adequate treatment was low (48.9%), it was higher than the proportion reported in other municipalities, as evidenced by Teixeira et al.^19^, in which only 20% of the women received adequate treatment. In order to ensure adequate and appropriate treatment, it is essential to form a bond between the health professional, the pregnant woman, and her partner, highlighting the importance of the primary care network and committed health teams. The low rates of treatment of women’s sexual partners could reflect problems with healthcare access among men; thus, the inclusion of men in antenatal care programs, in order to reduce the numbers of those who abandon treatment or receive inadequate treatment, is crucial.

Given that some pregnant women may not adhere to treatment, there is a need to improve the quality of care through tools such as the continuing education of professionals. This could be achieved by promoting programs such as the Permanent Education in Health, a program designed by the Ministry of Health that provides education to health professionals in their workplace, and aims to provide meaningful learning in order to transform professional practices in daily work^20^. In primary health care, active screening of pregnant women is essential to ensure that no cases are undiagnosed, and when syphilis is diagnosed, it is essential that all the items on the notification forms are filled-out accurately. This enables the profiles of the pregnant women with syphilis to be determined, thus making it possible to invest in appropriate methods of prevention. Continuing education should be provided to health professionals and the community, as knowledge regarding syphilis is fundamental for its prevention and control.

The low proportion of pregnant women in this study whose sexual partners were tested and treated for syphilis should not be attributed to the pregnant women alone, as men’s health-seeking behavior may have led them to resist treatment. Many men report feelings of fear, shame, carelessness, lack of time to seek care, and a lack of support by the health service system^21^ ^,^ ^22^. In addition, one study found that women were afraid of experiencing violence or being labeled as unfaithful if they disclosed their diagnosis of syphilis to their partners^23^. Thus, even if women are adequately treated, they may be re-infected by their sexual partners. This highlights the need to make treatment more accessible and welcoming among this population^6^ ^-^ ^15^ ^-^ ^21^.

We found a high incidence of congenital syphilis among the infants of women diagnosed with syphilis during pregnancy. Congenital syphilis is the leading cause of neonatal death and congenital malformation. The high incidence rate of congenital syphilis indicates a failure of the healthcare system, especially in primary care where there is the possibility of intervention^2^ ^-^ ^24^.

The recent increase in the syphilis diagnosed during pregnancy indicates that despite the high coverage, antenatal care is insufficient to prevent, diagnose, and treat this infection adequately. The inadequate treatment of pregnant women and their partners highlights the vulnerability of these women, mainly because syphilis is an STI. The majority of these women were in the first and second trimesters of pregnancy at the time of diagnosis, and thus had a high risk of reinfection due to inappropriate treatment, for themselves or their sexual partners. 

A limitation of this study is the amount of missing data in the disease notification forms. This highlights the need to improve the quality of reporting and case investigation. It also should be pointed out that it is possible that more male partners were treated than the number reflected in our data, but the deficiencies in the reporting methods could have led to them not being linked to their pregnant women partners. This study is likely to inspire research on syphilis during pregnancy, especially with respect to strategies to strengthen primary care. This will make it possible to achieve the goals related to the prevention of syphilis and a creating a better quality of life for pregnant women who are affected, as well as for their children.

There is need for continual improvement of the reporting system; and staff training should be carried out consistently in primary care. In addition, receptivity toward care and the formation of bonds between health professionals and primary care users is essential for successful care provision.

Finally, our results showed that social, economic, and cultural vulnerability influenced the occurrence of syphilis in different demographic groups of pregnant women, despite risk-reduction strategies on the part of the health services. The groups most affected by syphilis were the groups neglected based on their economic and social situations. 
